# High throughput biochemical profiling, and functional potential analysis for valorization of grape peduncles

**DOI:** 10.1038/s41598-023-34893-3

**Published:** 2023-05-23

**Authors:** Ali Akbar, Zareen Gul, Nadia Hussain, Amal H. I. Al Haddad, Nazir Ahmad Khan, Muhammad Bilal Sadiq, Hassan Sher

**Affiliations:** 1 Department of Microbiology, University of Balochistan, Quetta, 87300 Balochistan Pakistan; 2 Department of Botany, University of Balochistan, Quetta, Balochistan Pakistan; 3 Department of Pharmaceutical Sciences, College of Pharmacy, Al Ain University, Al Ain, UAE; 4 AAU Health and Biomedical Research Center, Al Ain University, Abu Dhabi, UAE; 5 Chief Operations Office, Sheikh Shakhbout Medical City (SSMC) in Partnership with Mayo Clinic, Abu Dhabi, UAE; 6 Department of Animal Nutrition, The University of Agriculture, Peshawar, Pakistan; 7 KAM School of Life Sciences, Forman Christian College (A Chartered University), Lahore, 54600 Pakistan; 8 Centre for Plant Sciences and Biodiversity, University of Swat, Charbagh, 19120 Khyber Pakhtunkhwa Pakistan

**Keywords:** Biochemistry, Biotechnology, Microbiology

## Abstract

*Vitis vinifera* L., commonly known as grape is a major fruit crop in the world. Grapes seem to confer health benefits due to their chemical components, biological and antioxidant activities. The present study is conducted to evaluate the biochemical constituents, antioxidant, and antimicrobial potential of ethanolic grape peduncles (EGP) extract. The result of phytochemical analysis revealed the presence of various phytochemicals such as flavonoid, tannin, carbohydrates, alkaloids, cardiac glycoside, phenol, steroid, terpenoids, quinones and anthraquinones. Furthermore, total phenolic content (TPC) and total flavonoid contents (TFC) were 7.35 ± 0.25 mg GAE/g (Gallic Acid Equivalent per gram) and 29.67 ± 0.13 mg QE/g (Quercetin Equivalent per gram) respectively. DPPH (2, 2-diphenyl-1-picrylhydrazyl) free radical scavenging assay revealed IC_50_ = 159.3 μg/mL. The antibacterial and antifungal study disclosed that the extract was highly potent against *Salmonella typhi* with maximum zone of inhibition of 27.2 ± 1.60 mm and *Epidermophyton floccosum* with 74 ± 1.81% inhibition. The extract was analyzed for its cytotoxicity and antileishmanial activity and showed no activity against HeLa cell line and promastigotes of *Leishmania major*. Elements Fe, Mn, Ni, Pb and Cd were determined by atomic absorption spectroscopy and approximately 50 compounds were identified by Gas Chromatography-Mass Spectrometry (GC–MS). Current work suggest that grape peduncles can be a promising source of bioactive medicinal component.

## Introduction

Since the beginning of time, humans have relied on plant-based sustenance for growth, development, energy and survival. Based on numerous research evidences, various societies all over the world have created their own folk medicinal systems that make use of their native flora^1^. Plants provide a variety of phytochemicals that have health benefits in addition to nutritional value and the potential for the creation of novel medications. In addition to balanced herbal foods, folk medicinal products are produced from edible plants that can be consumed as a single herb or in combination with other herbs in a variety of processed products and formulations^2^. The abundance of plants on the earth's surfaces has led to the growing interest in correlating phytochemical constituents of plants with their pharmacological activity. Alkaloids, flavonoid, tannin, glycoside, saponin, proteins and phenolic compounds are a few examples of the bioactive substances found in plants that are responsible for their therapeutic value and specific physiological effects on the body^3^. The prevalence of diseases has increased due to a number of factors, the primary one being oxidative stress, which is a serious issue that is brought on by free radicals and can have both positive and negative effects on a living thing, depending on their concentration^4^. Numerous plants are known to be screened for significant phytochemicals, biological and antioxidant activities^5^. *Vitis vinifera* L. (Grape) is a historically significant fruit plant commonly referred to as Angoor belongs to the important family Vitaceae. Grapes have around 60 or 70 different species and numerous varieties that are widely grown for fruit, juice and mostly for wine^6^. Due to the huge demand for fresh, dry, and wine production, grapes are produced all over the world, including Pakistan. Balochistan is where the majority of Pakistan's grape crop is produced, with the top three districts being Quetta, Pishin, and Mastung. The most well-known and well grown grape varieties are Kishmishi and Haita. Popular commercial cultivars Shundokhani, Sahibi and Shekhali are grown often in the districts of Quetta, Pishin, Mastung, Kalat, Killa Abdullah, Zhob and Loralai^7^. Even though the majority of its parts are beneficial, the grape is largely regarded as a source of distinctive natural ingredients that may be used to make a variety of industrial products as well as effective medications for a range of ailments^8^. Since ancient times, the fruit of the grapes as well as its seeds, leaves and skins have been employed in herbal medicine. Grape leaves have astringent and haemostatic effects and is high in flavonoid, tannin and procyanidin^9^. Grapes are full of nutrients and have significant amounts of glucose, fructose, sucrose, formic acid, citric acid, particularly malic acid, and tartaric acid. Hepatitis, varicose veins, haemorrhoids, inflammatory illnesses, diarrhea, and other conditions linked to free radicals are all treated with grape in the conventional medical system. The seeds and skins are rich in bioactive substances such as flavonoids, polyphenols, procyanidins, anthocyanin and resveratrol derivatives. In addition, grape has two major phenolic compounds including flavonoids which are made up of colorless flavan-3-ols, flavonols and anthocyanin and phenolic acids which include derivatives of cinnamic and benzoic acids^8^. These compounds are responsible for several pharmacological activities such as antibacterial, antifungal and anthelminthic, antioxidant, anti-inflammatory, hepatoprotective, antispasmodic and cytotoxic^5,10^. Additionally, the juice of the grape whose fruit can be white as well as its green, purple, or red leaves, which have antibacterial properties, is advised for use as eye wash. Numerous published research studies have offered evidence of traditional uses of grape and demonstrated a diversity of functions^11,12^. The chemical nature, phenolic and flavonoid content and antioxidant activity of seed, skin and stem of grapes and the wines made from them have all been the subject of studies. However, the investigations on the bioactive components, their chemical nature and pharmacological effects of grape peduncle are constrained. Therefore, determining the phytochemical components, total phenolic and flavonoid content, antioxidant capacity, trace elements, antibacterial and antifungal activity of ethanolic extract of *Vitis vinifera* (grape peduncle) was the goal of the current study in addition with using GC–MS profiling for the examination of significant bioactive components. The results of this study may help users to consume grapes and their peduncles for better health benefits.

## Results

The phytochemicals analysis of EGP extract determined that it contained various phytochemicals including flavonoid, tannin, alkaloids, cardiac glycoside, carbohydrates, phenol, steroid, quinones, terpenoids and anthraquinones while saponin and coumarin as were absent as mentioned in (Supplementary Table 1).

### Total phenolic and flavonoid contents determination of EGP

**Figure 1:**
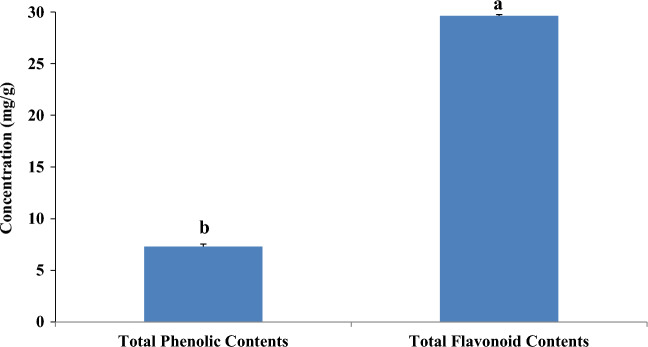
Total phenolic content expressed as mg GAE/g and Total flavonoid content expressed as mg QE/g of dried samples of grape peduncles (EGP). Bars represent the standard deviations of means. Different small superscript letters above the bars indicate means which are significantly different (p < 0.05).

The current analysis was to determine the total phenolic and flavonoid content of EGP extract using Folin-Ciocalteu sand aluminum chloride reagents. The analysis revealed that EGP extract contained the 29.67 ± 0.13mgQE/g dry weight of total flavonoid content and the total phenolic content was observed as 7.35 ± 0.25mgGAE/g dry weight (Supplementary Table 2). Statistically significant difference (P < 0.05) was noted in the mean Phenolic and flavonoids contents of EGP extract (Fig. 1).

### Antioxidant estimation of EGP extract by DPPH assay

**Figure 2:**
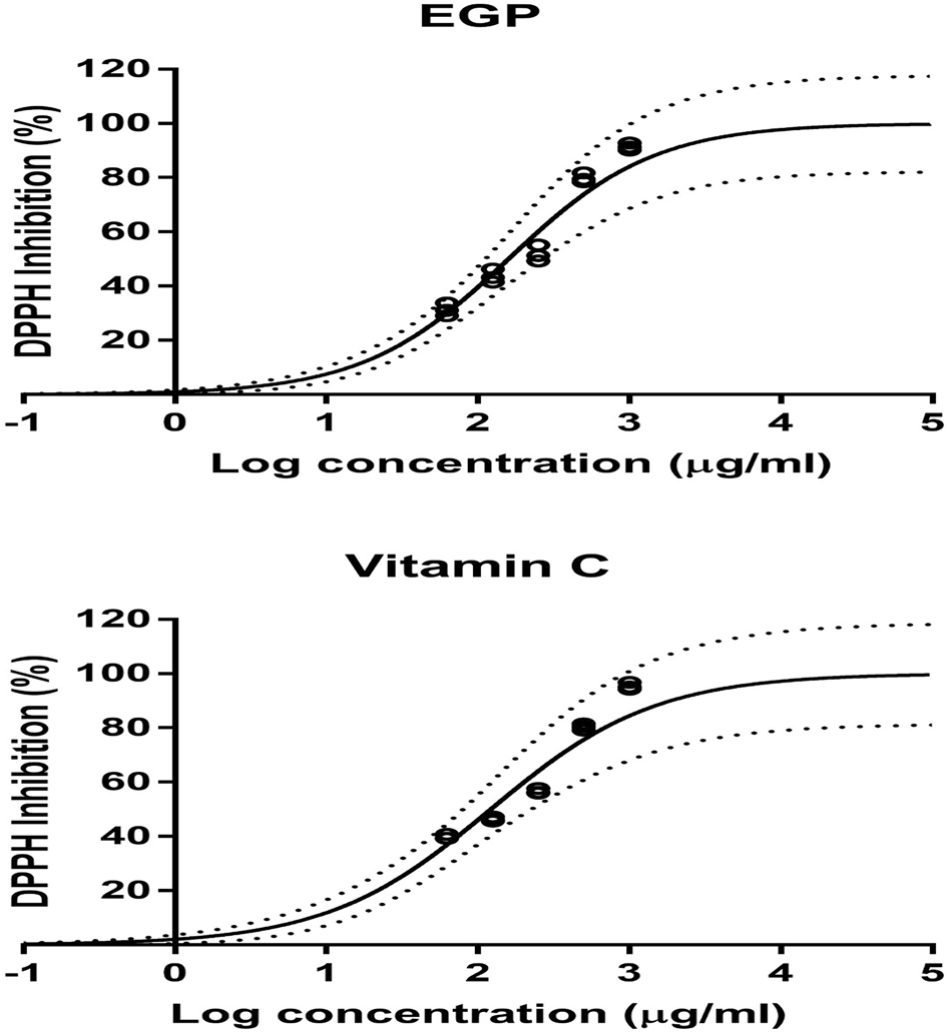
The antioxidant activity of EGP extract and standard Ascorbic acid (Vitamin C) at different concentrations, using non-linear regression. Open circles represent observations, the solid lines represent the estimated means curves, and the broken lines represent the 95% confidence intervals of the mean estimates.

This assay was conducted with synthetic DPPH which is widely used to evaluate scavenging activity of both natural and synthetic chemicals. The IC_50_ value represented the 50% antioxidant capacity. DPPH free radical scavenging activity and IC_50_ values are inversely correlated to each other (Fig. 2). The level of scavenging activity increases as the IC_50_ value decreases Table 1.

**Table 1:** The antioxidant percent inhibition and IC_50_ value of EGP extract and Ascorbic acid Parameters presented as mean estimates with 95% confidence intervals.

Samples	DPPH assay (IC_50_ μg/mL)
EGP	159.3 (138.8–185.5)
Ascorbic acid (standard)	121.8 (101.7–145.7)

### Antibacterial activity of EGP extract

The antibacterial activity of ethanolic grape peduncles (EGP) extract was determined by calculating the zone made around wells. Obtained results showed that EGP extract has remarkable antibacterial activity. The measured results were presented in (Table 2). The DMSO was taken as negative control in agar wells. The present study revealed that *Salmonella typhi* was a highly sensitive strain to the EGP extract with maximum zone of inhibition of 27.2 ± 1.6 mm among the other strains. The *Staphylococcus aureus* showed antibacterial potential with inhibition zone of 25.21 ± 1.2 mm. Similarly, *Klebsiella pneumoniae* and *Escherichia coli* were observed to inhibit bacterial growth with nearly equal inhibitory zones of 20.33 ± 2.51 mm and 22.15 ± 1.6 mm respectively. The results along with significance are illustrated in Table 2

**Table 2:** The antibacterial potential of EGP extract with inhibitory zones in mm as mean ± standard deviation.

Sample	Diameter of inhibition zone (mm)			
	*S. aureus*	*E. coli*	*S. typhi*	*K. pneumonia*
Ethanolic grape peduncles extract	25.21 ± 1.2^aAB^	22.15 ± 1.6^aBC^	27.2 ± 1.60^aA^	20.33 ± 1.51^aC^
Doxycycline	19.32 ± 0.25^bB^	23.41 ± 1.13^aA^	15.43 ± 0.9^bC^	21.14 ± 0.42^aB^

*S. aureus* = *Staphylococcus aureus*, *E. coli* = *Escherichia coli*, *S. typhi* = *Salmonella typhi*, *K. pneumoniae* = *Klebsiella pneumoniae.* Different small superscript letters within column indicate means which are significantly different (p < 0.05). Different capital superscript letters within rows indicate means which are significantly different (p < 0.05).

### Antifungal activity of EGP extract

**Figure 3:**
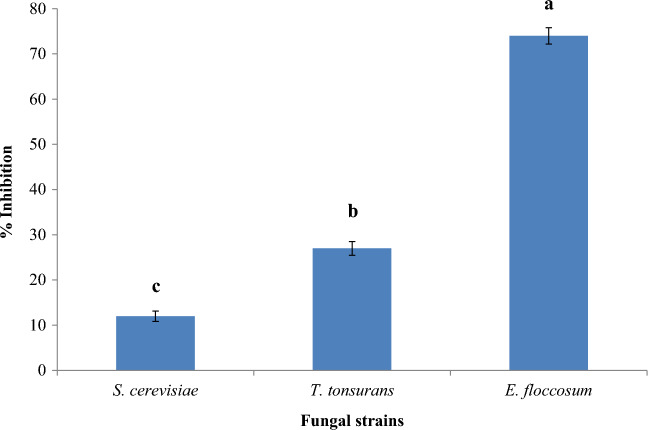
Antifungal activity of EGP extract against *S. cerevisiae, T. tonsurans* and *E. flocosum*. Here, *E. floccosum* = *Epidermophyton floccosum*, *S. cerevisiae* = *Saccharomyces cerevisiae*, *T. tonsurans* = *Trichophyton tonsurans.* Small superscript letters (a,b,c) indicate means which are significantly different (p < 0.05).

In the present investigation, the ethanolic extract of grapes peduncles (EGP) extract was tested against three fungal isolates and revealed that EGP extract inhibited *E. floccosum* with percent inhibition of 74 ± 1.81% while EGP was not active against *S. cerevisiae* 12 ± 1.15% and *T. tonsurans* 27 ± 1.52% (Fig. 3). The *E. floccosum* revealed a significantly higher (p < 0.05) level of inhibition compared with *T. tonsurans* and* S. cerevisiae.*

### Antileishmanial activity of EGP extract

The antileishmanial test was conducted to determine the antileishmanial activeness of promastigotes (*L. major*). A twofold serial dilution of the sample (1 mg/mL) was carried out in an assay. Pentamidine standard drug with (IC_50_ = 3.16 ± 0.15 mg/mL) was utilized to compare the parasite inhibition by EGP extract. The IC_50_ value observed for extract against *L. major* was (IC_50_ ≥ 100 mg/mL) and exposed no antileishmanial effect as showed in (Table 3). According to results, Pentamidine decreased the viability of promastigotes in all concentrations by investigating with each extract.

**Table 3:** Estimated IC_50_ values of ethanolic grape peduncle (EGP) extract against Promastigotes and HeLa cell line.

Samples	IC_50_ (mg/ml) ± SD promastigotes	IC_50_ (mean ± SD, µg/ml) HeLa cell line
EGP	> 100	> 100
Pentamidine	3.16 ± 0.15	–
Doxorubicin	–	09 ± 0.14

Pentamidine and Doxorubicin = control.

### Anticancer activity of EGP extract

The MTT cell assay was used to assess the anticancer activity of the EGP extract against the HeLa cell line. Doxorubicin was employed as the reference drug to compare the inhibitory effects of the extract and the experiment was carried out at 1 mg/mL of extract. Doxorubicin showed stronger anticancer activity (IC_50_ = 09 ± 0.14 mg/mL) than the EGP extract (IC_50_ ≥ 100 mg/mL) which had no anticancer effect. According to results, Doxorubicin decreased the viability of HeLa cell line in all concentrations by investigating with each extract (Table 3).

### Elemental composition of grape peduncles

**Figure 4:**
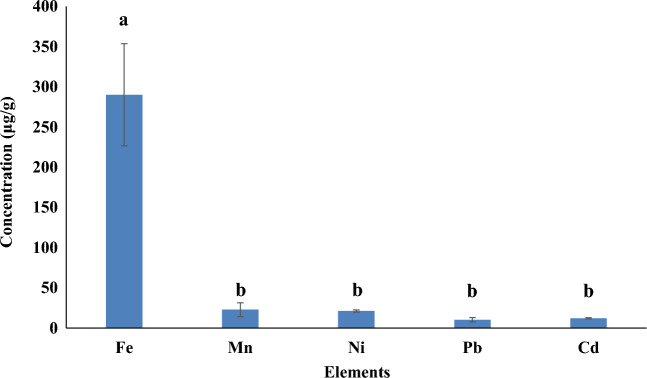
Comparative analyses of various metals concentrations in grape peduncles, Here Fe = Iron, Mn = Manganese, Ni = Nickel, Pb = Lead, Cd = Cadmium. Different small superscript letters (a,b) indicate means which are significantly different (p < 0.05).

Trace element concentration analysis is extremely important regarding the quality control of food and diet. The analysis of Fe, Cd, Mn, Ni and Pb were done by atomic absorption spectrometry. The present study evolved that Fe was present in concentration of 290.13 ± 63.52 μg/g while Mn content was 22.86 ± 0.82 μg/g. Similarly, Nickel contents were 21.26 ± 1.41 μg/g, whereas Pb was present in low concentration of 10.37 ± 0.14 μg/g and Cd was 12.14 ± 1.02 μg/g. The data is significantly different for each element (p < 0.05) and the results are presented in (Fig. 4).

### Gas chromatography mass spectrometry (GC–MS) analysis

Using GC–MS, nearly 50 different compounds were identified and shown in (Table 4) along with their retention times, molecular formula, molecular weight and Area%. From the obtained results the grape peduncle extract contained various components including alcohol, ether, amide, fatty acids and heterocyclic compounds. The major bioactive constituents were Tetramethyl silicate, 2,2-Dimethoxybutane, Dihydro-2(3H)-thiophenone, Glycolaldehyde dimethyl acetal, 3-Methyl-oxirane-2-carboxylic acid, methyl ester, Butanoic acid, 2-hydroxy-, methyl ester, 3-Methylbenzyl alcohol, TBDMS derivative, 3-Methylbenzyl alcohol, 2-Furanmethanol, 1-Butanol, 2-amino-3-methyl-, Methyltrioxitol, 1,2-Cyclopentanedione, 3-Bromobutyric acid, Thietane, 1-oxide, Carbonochloridic acid, ethyl ester, Ethyl (trimethylsilyl)acetate, Erythritol, Ethane, 1-chloro-1-fluoro-, Oxalyl chloride, Vanillic Acid, 3-Deoxyglucose, Glycerin, D-erythro-Pentose, 2-deoxy, Ethoxydi(tert-butyl)silane, 3-Methoxybenzyl alcohol, methyl ether, Arachidonic acid, TMS derivative, Acetic acid, [4-methoxy-3-(trimethylsiloxy) phenyl], Pyrogallol, Hydroquinone, Prednisone, Silane, triethoxymethyl-, o-Cymene, Rhamnitol, 1-O-nonyl, l-(+)-Lactic acid, 2TMS derivative, Neophytadiene, Z-3-Octadecen-1-ol acetate, Imidazole-2-[3-thiopropionic acid], Stannane, butyltrimethyl-, Thymol, Trinexapac-ethyl, Silanol, trimethyl-, phosphite (3:1), Carvacrol, TBDMS derivative, Diisooctyl phthalate, Vitamin E, Hexadecanoic acid, methyl ester, 3,4-Dihydroxymandelic acid, 9,12-Octadecadienoic acid (Z,Z)-, Oleic Acid, Ergostane-3,5,6,12,25-pentol, 25-acetate, Tetracosamethyl-cyclododecasiloxa, Hexasiloxane tetradecamethyl respectively.

**Table 4:** Major compounds analyzed in the ethanolic grape peduncles EGP extract by GC–MS analysis.

S#	R.T (min)	Area (%)	Name of the compound	M. Formula	M. wt
1.	3.023	0.03	Tetramethyl silicate	C_4_H_12_O_4_Si	152
2.	3.075	0.01	2,2-Dimethoxybutane	C_6_H_14_O_2_	118
3.	3.111	0.01	Dihydro-2(3H)-thiophenone	C_4_H_6_OS	102
4.	3.179	0.02	Glycolaldehyde dimethyl acetal	C_4_H_10_O_3_	106
5.	3.383	0.02	3-Methyl-oxirane-2-carboxylic acid, methyl ester	C_5_H_8_O_3_	116
6.	3.424	0.02	Butanoic acid, 2-hydroxy-, methyl ester	C_5_H_10_O_3_	118
7.	3.553	0.07	3-Methylbenzyl alcohol, TBDMS derivative	C_14_H_24_OSi	236
8.	3.596	0.01	2-Furanmethanol	C_5_H_6_O_2_	98
9.	3.952	0.04	1-Butanol, 2-amino-3-methyl-,	C_5_H_13_NO	103
10.	4.028	0.03	Methyltrioxitol	C_10_H_24_O_4_Si	236
11.	4.270	0.04	1,2-Cyclopentanedione	C_5_H_6_O_2_	98
12.	4.341	0.05	3-Bromobutyric acid	C_4_H_7_BrO_2_	166
13.	4.526	0.08	Thietane, 1-oxide	C_3_H_6_OS	90
14.	4.815	0.08	Carbonochloridic acid, ethyl ester	C_3_H_5_ClO_2_	108
15.	4.933	0.56	Ethyl (trimethylsilyl)acetate	C_7_H_16_O_2_Si	160
16.	5.182	0.25	Erythritol	C_4_H_10_O_4_	122
17.	5.699	0.34	Ethane, 1-chloro-1-fluoro-	C_2_H_4_ClF	82
18.	5.850	0.32	Oxalyl chloride	C_2_C_l2_O_2_	126
19.	5.865	0.07	Vanillic Acid	C_14_H_24_O_4_Si_2_	312
20.	6.667	0.03	3-Deoxyglucose	C_6_H_12_O_5_	164
21.	7.143	0.06	Glycerin	C_3_H_8_O_3_	92
22.	7.670	0.08	D-erythro-Pentose, 2-deoxy	C_5_H_10_O_4_	134
23.	7.975	0.04	Ethoxydi(tert-butyl)silane	C_10_H_24_OSi	188
24.	8.565	0.03	3-Methoxybenzyl alcohol, methyl ether	C_9_H_12_O_2_	152
25.	9.040	0.03	Arachidonic acid, TMS derivative	C_23_H_40_O_2_Si	376
26.	9.760	0.08	Acetic acid, [4-methoxy-3-(trimethylsiloxy)phenyl]	C_16_H_28_O_5_Si_2_	356
27.	10.285	0.04	Pyrogallol	C_15_H_30_O_3_Si_3_	342
28.	11.775	0.06	Hydroquinone, 2TMS derivative	C_20_H_38_O_2_Si_2_	366
29.	12.040	0.08	Prednisone	C_21_H_26_O_5_	358
30.	13.465	0.06	Silane, triethoxymethyl	C_7_H_18_O_3_Si	178
31.	13.499	0.08	o-Cymene	C_10_H_14_	134
32.	15.080	0.03	Rhamnitol, 1-O-nonyl	C_15_H_32_O_5_	292
33.	15.901	0.04	l-(+)-Lactic acid, 2TMS derivative	C_9_H_22_O_3_Si_2_	234
34.	16.681	0.04	Neophytadiene	C_20_H_38_	278
35.	17.070	0.03	Z-3-Octadecen-1-ol acetate	C_20_H_38_O_2_	310
36.	17.940	0.08	Imidazole-2-[3-thiopropionic acid]	C_6_H_6_BrN_3_O_4_S	295
37.	18.023	0.01	Stannane, butyltrimethyl	C_7_H_18_Sn	222
38.	20.665	5.35	Thymol	C_10_H_14_O	150
39.	26.060	0.08	Trinexapac-ethyl	C_16_H_24_O_5_Si	324
40.	27.065	0.02	Silanol, trimethyl-, phosphite (3:1)	C_9_H_27_O_3_PSi_3_	298
41.	29.565	0.07	Carvacrol, TBDMS derivative	C_16_H_28_OSi	264
42.	30.847	0.12	Diisooctyl phthalate	C_24_H_38_O_4_	390
43.	41.018	2.01	Vitamin E	C_29_H_50_O_2_	430
44.	41.910	0.46	Hexadecanoic acid, methyl ester	C_16_H_32_O_2_	256
45.	46.155	0.52	3,4-Dihydroxymandelic acid,	C_20_H_40_O_5_Si_4_	472
46.	49.398	1.89	9,12-Octadecadienoic acid (Z,Z)	C_19_H_34_O_2_	294
47.	49.974	4.76	Oleic Acid	C_18_H_34_O_2_	282
48.	50.920	0.04	Ergostane-3,5,6,12,25-pentol, 25-acetate,	C_39_H_76_O_6_Si_3_	724
49.	54.517	0.34	Tetracosamethyl-cyclododecasiloxa	C_24_H_72_O_12_Si_12_	888
50.	58.740	0.08	Hexasiloxane tetradecamethyl	C_14_H_42_O_7_Si_7_	518

*M. Formula* Molecular Formula, *M. Wt* Molecular Weight, *RT* Retention time.

## Discussion

Plants exhibit biological characteristics such as antioxidant effects, antibacterial properties, detoxification of enzymes, immune system activation, reduction of platelets, activation of hormones biosynthesis and anticancer capabilities. These biological effects of plants have been strongly associated with their biologically active substances. One of the most delicious and high-quality plants is the grape (*Vitis vinifera *L.), which is a member of the Vitaceae family. Numerous nutraceuticals including resveratrol, are derived from grapes, which may have health benefits for the cardiovascular system, cancer chemo preventive potential, skin cancer prevention and treatment and other less common but deadly diseases like Alzheimer's and urinary bladder dysfunction. Additionally, the plant helps improve liver and heart functioning by reducing liver damage and treating cardiovascular disease^13^.

The ethanolic grape peduncles extract was screened for the presence of major secondary metabolite classes according to common phytochemical methods. The tests relied on visual examination of the color change or precipitate deposition following the addition of a particular reagent. All the identified components in the current investigation are known to have a diverse range of biological effects. Compared with previous studies Hanaa et al.^14^ reported the occurrence of steroids, anthocyanins, terpenoid, glycoside, flavonoid and phenols. Previously, flavonoid, procyanidins, anthocyanin, polyphenols and resveratrol derivative were reported in seeds, pulp and skin of grapes^8^. These phytochemicals are known to confer certain health benefits such as anti-inflammatory, anti-hypertensive, antimicrobial and anti-diabetic property^15^.

Due to their redox properties, phenolic and flavonoid contents which are abundant in plants have been discovered to have antispasmodic, antibacterial, anti-inflammatory, anticancer, antifungal, antidepressant and antioxidant power^16^. Phenolic compounds regulate plant cell division and development, also stabilize lipids against peroxidation and neutralize metabolic processes by scavenging free radicals. The variations in flavonoid structure and their modifications affect the phenoxyl radical stability, which in turn affects its antioxidant capabilities. A study conducted by Doshi et al.^11^ revealed total phenolic contents (96.7 mg/ml) and flavonoid (31.5 mg/ml) in grape byproducts which are contrary to current findings. The TPC and TFC values were positively correlated to the documented results of Khan et al.^17^. Obtained results were also in agreement with the^18^. When compared to previous literature^19^, higher phenolic contents were observed in grape peel. Another study revealed highest phenolic (392.58 ± 1.70 mg of GAE/g) and flavonoid (256.16 ± 1.60 mg of QE/g) contents in grapes seed as compare to our study^20^. Our findings were also in conformity with the referenced results of Rani et al.^21^. Understanding the composition and synthesis of such molecules which have a specialized role to restrict and curtail the several pathogen attacks and diseases induce in it, would be of extreme interest in the ecological role of grape phenolic and flavonoid compounds and their antioxidants activities. Grape peduncles are thought to be a significant source of bioactive compounds and play a significant role in the production of components for nutraceuticals, medications, and foods.

Numerous studies have shown that the therapeutic properties of medicinal herbs, fruits and vegetables are due to the presence of different phytochemicals with antioxidant activity^22^. Current results were in accordance with the DPPH radical scavenging properties in fresh and dried grape skin, pulp and seed with higher percent antioxidant activity^21^. The findings were also in line with earlier research in which the antioxidant capabilities of different fruits were evaluated^18^. Recently Khan et al.^17^ documented the strong antioxidant power of leaves of grape varieties by DPPH radical scavenging assay. The antioxidant activity of organic grape peel was also calculated by using DPPH radical scavenging with higher percent of inhibition^19^.

Using the agar well diffusion method, the antibacterial effect of extract was assessed. The bacterial species *E. coli, S. typhi, K. pneumonia* and *S. aureu*s were all thoroughly countered by the EGP extract. The zone of inhibition measures for the tested agents were noted in mm and as given in Suppl. Table 1. The zones of inhibition with 10–16 mm is referred average, higher than 16 mm are active and those less than 10 mm were regarded weak. Obtained results showed that EGP extract has significant antibacterial activity. The overall measured results revealed that *Salmonella typhi* is a highly sensitive strain to the EGP extract with maximum zone of inhibition followed by *Staphylococcus aureus,* The Bacterial strains *Escherichia coli and Klebsiella pneumoniae* respectively. According to the reported results of Ali et al.^23^, the methanolic grape leaf extract showed strong antibacterial effect against infectious bacterial strains *Staphylococcus aureus*, *Streptococcus viridans*, *Clostridium septicum* and *Escherichia* coli with highest zones of inhibition. Current results were in accordance with results of Ahmad et al.^24^. The remarkable antibacterial potential of grape pomace against bacterial strains *Escherichia coli* and *Staphylococcus aureus* were also reported by Saratale et al.^25^.

Billions of people are infected by fungus each year. Regardless of the substantial rates of infection and mortality, fungal infections are still little understood. Conventional drugs are not particularly efficient in treating fungal disorders and not many products are being developed presently. Additionally, several studies claim that flavonoids are efficient antifungal compounds against a variety of pathogens. Grape pomace fluid extract had considerable potency against the Candida species *C. albicans*, *C. krusei* and *C. parapsilosis*^26^. Other study by Khan et al.^27^ revealed effective antifungal activity of grape leaves extracts against *Penicillium expansum.* In current study the ethanolic grape peduncles EGP extract was analyzed by agar mix method and resulted in potent antifungal activity against three fungal strains *E. floccosum*, *S. cerevisiae* and *T. tonsurans.*

One of the most significant vector-borne diseases, leishmaniasis is indigenous to tropical and subtropical regions. There are numerous clinical strategies that have been approved for the various forms of leishmaniasis, but their usage is all constrained by unfavorable side effects. Here, we aim to assess the EGP extract for in vitro antileishmanial effectiveness on *L. major*, but the extract showed no antileishmanial effect. In previous study Mansour et al.^28^ documented the effect of *Vitis vinifera* L. leaves extract on *Leishmania infantum.* Another study revealed the antileishmanial activity of flavonoid against *L. donovani*^29^. Another study reported by^30^ disclosed the strong antileishmanial potential of plants against *Leishmania tropica.*

Cancer is a widespread public threat with an estimated 6 million new cases globally each year. It ranks after cardiovascular disorders as the second leading cause of death. The term "cancer" refers to a group of malignant disorders that can affect diverse physiological systems. Unregulated and rapid development of malignant tumors, which can aggregate to form a growth or propagate all through the body and cause abnormal growth, is a hallmark of many disorders. It can continue until the organism perishes^31^. Consuming a lot of fruits and vegetables can help to reduce the occurrence of cancer since several naturally occurring components in human diet have been identified as potential chemo-preventive agents. It has been shown that a variety of organic compounds have powerful anticancer effects against several rodent and human cancer cell lines. Using the MTT experiment on HeLa cell lines, EGP extract had no cytotoxic effect. In a study conducted, cytotoxic activity against HeLa cell lines and MCF7 cell lines was examined using various plant extracts in different solvents. The extract exhibited lower efficacy against HeLa cell lines but exhibited remarkable activity against MCF7 cell lines^32^.

Due to its high resolution, narrow detection wavelengths, simplicity of use and lower acquisition requirement, atomic absorption spectroscopy (AAS) is an effective method for measuring elements. It is mostly vulnerable, and the number of different substances can only be measured at a low level. The main benefit and widespread application of this technology is for elements assessment. One of the most crucial substances known to produce red blood cells in the body is iron. It is a crucial micro-nutrient for all sentient creatures. Its low levels cause anemia, and its high levels harm the body tissues. Iron is generally not regarded to be damaging to health, save for when it is ingested in toxic concentrations. Iron levels in plants should not exceed 20 mg/kg while human consumption levels range from 10 to 28 mg/day^33^. The obtained Fe contents 290.13 ± 63.52 µg/g in grape peduncles indicate the possibility of treating iron deficiency with grape. Manganese is a vital component for growth and a superior antioxidant. In grape peduncles Mn resulted as 22.86 ± 0.82 µg/g while the needed range for Mn is 20 mg/kg for eatable plants. According to literature, an appropriate daily intake of manganese is between 3 and 6 mg, with a maximum of 12 mg per day^34^.

Nickel controls a variety of metabolic processes in plants. Loss of body weight, liver issues, and heart dysfunction are all consequences of its inadequacy. When nickel levels are high, plants experience severe chlorosis, necrosis, and anatomical alterations. The obtained amount of Ni from grape peduncles is 21.26 ± 1.41 μg/g and daily intake for Ni is 14 μg/day for human being^33^. Even though lead is widely distributed in our surroundings, it does not constitute a toxic but non-essential element. Its applications make it unique in terms of ecological hazard, so understanding is necessary for comprehending toxic substances^35^. In grape peduncles the concentration of lead was 10.37 ± 0.14 μg/g, in contrast to the acceptable levels in curative plants, which are 10 mg/l, the safe level of lead for human usage is 1.5 mg/l. Cadmium is a non-essential heavy metal that is toxic for human health even in very low concentration. Considering the permissible limit of Cd being 0.3 mg/kg for edible plants the obtained results 12.14 ± 1.02 μg/g indicated the level of this element in grape peduncle. However, result was in agreement with the Cd concentration ranges previously reported^36^.

To quantify the important chemicals found in different grape samples, Gas chromatography and mass spectroscopy (GC–MS) has been the preeminent analytical approach in combination with a variety of sample preparation protocols. The obtained results show that the mass spectra have been used to identify 50 distinct compounds. From the obtained results the grape peduncles extract contained various components including alcohol, ether, amide, fatty acids, and heterocyclic compounds. According to Kadhim et al.^37^ 2-Furanmethanol has the strong anti-inflammatory activity which was isolated from methanolic extract of grape. Hexadecanoic acid has potent antimicrobial activity and Hexasiloxane tetradecamethyl is a strong anticancer agent while Octadecadienoic acid (Z, Z)—has powerful anti-inflammatory, and anti-diabetic power^16^. Carvacrol derivative has high as antimicrobial, antimutagenic, anti-inflammatory, antitumor, antiparasitic activity^38^. Oleic acid is a putative antioxidant that is formed in foods including sugar. Oleic acid is an abundant unsaturated fatty acid with pharmacological use as an insecticide, fungicide, and herbicide. Applications for oleic acid include medicines, food additive and emulsifier, while lactic acid, 2TMS derivative used as antibacterial, keratolytic and emollient agent in various cosmetic material^39^. Erythritol is a sweet antioxidant releases oxidative stress and has unique nutritional effects^16^. The significant biological and pharmacological effects of resulted compounds have already been documented by Rani et al.^32^. The current study showed that grape peduncles can be employed as a potentially useful multifaceted medicinal product, although additional clinical trials are needed to demonstrate their effectiveness.

## Conclusion

The findings indicated that grape peduncles extract is rich in phytochemical substances that have the ability to serve as antibacterial, antifungal and antioxidant agents. It was discovered that the extract has a substantial amount of phenolic and flavonoid contents with powerful antioxidant properties. Due to the presence of significant chemical compounds with strong biological potentials, recent findings show that the grape peduncles may be a promising source of active pharmaceuticals. The results of the research may inspire individuals to employ it as functional ingredients for humans, in wide range of diseases.

## Methods

### Sample collection and preparation

Grapes (*Vitis vinifera* L.) were collected from the local fruit market of district Pishin, Balochistan. Grape’s peduncles were gently removed, decontaminated, washed with distilled water, and dried in shade at room temperature for 2–3 weeks. For further analysis, the dried peduncle was ground into fine powder and stored in a desiccator for extract making.

The research work and the Plant collection permission has been granted by the local and institutional authorities (No. UoB/Reg:/GSO/464 dated 01/06/2021).

### Extraction by maceration

In maceration extraction, 150 g of grapes peduncle were soaked in 1.5 l ethanol in ratio of 1:10 for three days, as per explained by Akbar et al.^3^. The process was carried out in the dark and was shaken at a specific time. The ethanolic extracts were filtered through Whatman filter paper No. 1. The filtrate was concentrated through rotary evaporator then the crude extract was before used for further chemical and biological profiling. Each analysis was conducted three times and experimental errors were kept at 5% or less of the mean values.

### Phytochemicals analysis of EGP

The qualitative analysis of phytochemicals included carbohydrates, alkaloids, cardiac glycoside, coumarin, saponin, steroids, tannin, quinine, terpenoid, flavonoid and phenol in EGP extract was carried out by the methodology explained by Gul et al.^16^.

### Total phenolic content (TPC) determination

TPC of EGP extract was estimated by following the procedure adopted by Tian et al.^40^. An amount of 0.1 of EGP extract was reacted with 0.5 ml of Folin Ciocalteu reagent and 0.6 ml of Na_2_CO_3_ (DAEJUNG Chemicals & Metals Co. Ltd Korea) (20% w/v) was added after 10 min of incubation. The reaction mixture was further incubated for 30 min at 40 °C. The absorbance of reaction mixture was measured at 765 nm using UV–VIS spectrophotometer (T60 PG, UK). 95% ethanol was taken as blank. The calibration curve for the calculation of phenolic compounds in sample was prepared by different dilution of Gallic acid (1 to 0.0625 mg/ml). The results were presented as mg GAE/g of sample dried weight.

### Total flavonoid content (TFC) estimation

TFC of EGP extract was calculated using the colorimetric technique with AlCl_3_ reagent by the methodology explained by Gul et al.^41^. The extract and standard dilutions of around 500 µl was added in 2.35 ml distilled water and 150 µl of NaNO_2_ solution (5%). After incubation, 150 µl of AlCl_3_·6H_2_O (10%) was added in incubated mixture. The 0.5 ml of (1 M) NaOH was added after 5 min. The final volume of reaction mixture was adjusted with distilled water up to 5 ml and then the absorbance was taken at 415 nm. The calibration curve was produced using Quercetin different dilutions ranging from 1 to 0.0625 mg/ml. The results were presented as mgQE/g of sample dried weight.

### Antioxidant activity of EGP

The antioxidant activity of ethanolic extract of grape peduncle EGP was measured by reacting DPPH radical with extract. The 0.1 mM DPPH solution was prepared using 100 ml of absolute ethanol and 0.0039432 g of DPPH using weighing balance (G&G JJ224BC 220 g/0.1 mg). The extract 50 μl was mixed with 0.5 mL of DPPH (0.1 mM) solution and incubated at 37 °C for 30 min in dark. Ascorbic acid (DAEJUNG Chemicals & Metals Co. Ltd Korea) was used as reference standard. The decolorization of mixture was taken at 517 nm. Ethanol was used as blank and DPPH was used as control respectively^41^. The scavenging capacity was calculated using the formula shown below.

$$\% {\text{ Scavenging }}\;{\text{activity}} = \left( {{\text{A1}} - {\text{A2}}} \right)/{\text{A1 }} \times { 1}00.$$

Absorbance of control = A1, Absorbance of extract = A2.

### Antibacterial activity

The antibacterial activity of EGP extract was conducted using agar well diffusion method opted by^42^. The target bacterial isolates (*Klebsiella pneumonia, Escherichia coli, Salmonella typhi and Staphylococcus aureus)* were refreshed in sterilized nutrient broth and incubated at 37 °C for 24 h. The 6 mm wells were made by sterile cork borer in sterile Muller Hinton Agar (Oxoid, UK) media plates and extract was put inside the wells. The plates were incubated for 24 h at 37 °C. Media having organisms was used as positive control, dimethyl sulfoxide was taken as negative control and Doxycycline as a reference drug respectively. The diameter of the clean zone around the wells were measured in millimeters (mm) and was used to interpret the findings.

### Antifungal activity

The antifungal activity of EGP extract was analyzed by the agar mix method following^43^. Briefly, 1.5 ml of extract was added to 25 ml of freshly made potato dextrose agar and mixed thoroughly. The media was poured into petri plates. Fresh cultures of the fungal strains (*Trichophyton tonsurans*, *Epidermophyton flocosum*) and yeast (*Saccharomyces cerevisiae*) were placed into 6 mm wells and incubated for 72 h at 37 °C. As positive and negative control, media with and without fungal strains were employed. After incubation, the growth inhibition of tested fungal strains was estimated by taking measurement of linear growth in millimeter (mm) regarding the negative control. The percent inhibition of extract was calculated using the following equation:

$$\% {\text{ Inhibition}} = \left( {{1}00 - {\text{linear }}\;{\text{growth }}\;{\text{in }}\;{\text{test }}/{\text{linear }}\;{\text{growth}}\;{\text{ in }}\;{\text{control}}} \right) \, \times { 1}00.$$

### Antileishmanial activity

The preserved culture of *Leishmania major* promastigotes were refreshed in Novy–MacNeal–Nicolle medium (NNN) with streptomycin and penicillin. The method earlier reported by^22,30^ was used for antileishmanial assay with few modifications. In this test, promastigotes in log phase at a concentration of 1 × 10^6^ cells/ml were used. The ethanolic grape peduncles EGP extract was tested against the promastigotes of *L. major* in 96-well plate. Each sample was serially diluted concentration of 1–0.062 mg/ml. Following the procedure, each well was filled with 50 µl of the promastigotes culture and 10µL of each sample dilution. Pentamidine was used as a standard drug. As positive and negative controls, media containing an organism and DMSO were taken, respectively. The plate was incubated for 72 h at 37 °C. Around 1 µl DMSO and 20 µl NBT solution was additionally added to each well. The absorbance was taken using microplate reader (RT-6000) at 630 nm. Using the linear regression approach, the IC_50_ values of extract were determined. The following formula given below was used to compute the percentage inhibition:

$$\% {\text{ Cell }}\;{\text{viability}} = \left( {{\text{A63}}0 \, \;{\text{of}}\;{\text{ test }}\;{\text{sample}}/{\text{A63}}0 \, \;{\text{of}}\;{\text{ control}}} \right) \, \times { 1}00.$$

$$\% {\text{ Inhibition}} = {1}00 - \% {\text{ viability}}.$$

### Anticancer activity

The anticancer activity of ethanolic grape peduncles (EGP) extract was observed by a colorimetric assay using MTT 3-(4, 5-dimethylthiazol-2-yl)-2, 5-diphenyltetrazolium bromide. The HeLa cell line were grown in a minimally medium supplemented with 10% FBS, 100 units/mL penicillin and 100 µg/ml of streptomycin at 37 °C in a humidified environment^44^. Concisely, 200 µl of cell mixture (1 × 10^6^ cell/ml) was grown in 96-well plates with incubation at 37 °C treatment of 100 µg/ml of extract for 48 h. The cell viability was evaluated by dissolving MTT in phosphate-buffered saline (PBS, pH 7.2), with addition of 20 µl (6 mg/ml in phosphate buffer) of 0.4% 3-(4, 5-dimethylthiazol-2-yl)-2, 5-diphenyltetrazolium bromide (MTT solution) followed by 4 h incubation. The magnitude of formazan defined was ascertained by taking the absorbance at 570 nm with a UV–VIS spectrophotometer (T60 PG, UK). DMSO was utilized as negative control and Doxorubicin was taken as standard. A standard curve was plotted, and results were calculated as the concentration required for 50% inhibition (IC_50_).

### Atomic absorption spectroscopy of trace elements

The analyses of Fe, Cd, Mn, Ni and Pb were determined through atomic absorption (Perkin–Elmer 3100 USA) spectrophotometer. The digestion of the sample was performed using a previous reported procedure^33^. After wet digestion, these produced solutions were examined for the elemental detection using atomic absorption spectroscopy (AAS). The study was carried out using deionized water. Each sample analyzed includes blanks for quality assurance and quality control purposes. For the analysis of every element, hollow cathode lamps and acetylene air flames were used as radiation source and as fuel and the wavelengths were set as 248.3 nm, 228.8 nm, 279.5 nm, 232 nm, and 216.9 nm for Fe, Cd, Mn, Ni and Pb respectively. The results were expressed as (μg/g) of sample.

### Gas chromatography-mass spectrometry

The ethanolic extract of the grape peduncle EGP was subjected to analysis on a GC–MS (Shimadzu GC–MS QP2020 Japan) supplied with a (HP-INNOWAX) capillary column with a dimension of 20 m × 0.18 mm, 0.18 mm film thickness (PaloAlto, CA, USA) running in electron impact mode at 70 eV; 0.5 s of scan interval and fragments from 40 to 550 Da using a mass scan frequency spectrum of 35–300 (atomic mass unit). Helium was used as carrier gas at a constant flow of 1 ml/min and an injection volume of 2.00 µl was employed (split ratio of 10:1); injector temperature 280 °C. The oven temperature was programmed from 30 °C (isothermal for 3 min), with an increase of 5 °C/min to 175 °C, then ending with an isothermal for 15 min at 200 °C. Injection mode was split-less and total GC running time was 70 min. Interpretation of mass spectrum GC–MS was done using the database of National Institute of Standard and technology (NIST) and for the identification of structure of the components, their names, molecular formula and molecular weight, Mass spectrum was used^45^.

### Statistical analysis

All the analyses for each finding of total phenolic contents, total flavonoid contents, antioxidant, biological and chemical attributes were conducted in triplicates. The values are stated as the mean ± standard deviation (SD) calculated by using Microsoft Excel Software and one-way analysis of variance (ANOVA) and Duncan’s multiple range tests were carried out to determine significant group differences (p < 0.05) between means by using SPSS statistical software package (SPSS, version 16.0).

### Statement regarding plant collection

The plant part collection and use were in accordance with all the relevant guidelines.

## Supplementary Information

Supplementary Tables.

## Data Availability

The data will be made available from the corresponding author on reasonable request.
