# Supplementary material for: High throughput biochemical profiling, and functional potential analysis for valorization of grape peduncles
Source: Sci Rep. 2023 May 23;13:8328. doi: 10.1038/s41598-023-34893-3 (PMC10206094; doi:10.1038/s41598-023-34893-3)
Supplement: Supplementary file 1 — Supplementary Tables. [file 41598_2023_34893_MOESM1_ESM.docx]

**Supplementary Table 1:** Phytochemicals analysis of ethanolic grape peduncles (EGP) extract

| Phytochemicals Constituents | EGP extract |
| --- | --- |
| Flavonoid | **+** |
| Tannin | **+** |
| Alkaloids | **+** |
| Cardiac glycoside | **+** |
| Carbohydrates | **+** |
| Saponin | **-** |
| Phenol | **+** |
| Steroid | **+** |
| Quinones | **+** |
| Coumarins | **-** |
| Terpenoids | **+** |
| Anthraquinones | **+** |

**Note**: EGP =Ethanolic extract of Grape Peduncle, (+) = Presence, (-) = Absence

**Supplementary Table 2** Total phenolic contents and Total flavonoid contents in ethanolic grape peduncles (EGP) extract

| **Samples** | **Total phenolics**  **(mg GAE/g dry sample)** | **Total flavonoid**  **(mg QE/g dry sample)** |
| --- | --- | --- |
| **EGP** | 7.35^a^ ± 0.25 | 29.67^b^ ± 0.13 |

**Note**: Results are expressed as Mean± S.D for three readings. Significant (p < 0.05) differences between groups are indicated by small superscript letters.
